# A Model-based Assessment of Oseltamivir Prophylaxis Strategies to Prevent Influenza in Nursing Homes

**DOI:** 10.3201/eid1510.081129

**Published:** 2009-10

**Authors:** Carline van den Dool, Eelko Hak, Marc J.M. Bonten, Jacco Wallinga

**Affiliations:** University Medical Center, Utrecht, the Netherlands (C. van den Dool, E. Hak, M.J.M. Bonten, J. Wallinga); University Medical Center, Groningen, the Netherlands (E. Hak); National Institute for Public Health and the Environment, Bilthoven, the Netherlands (J. Wallinga); 1 These authors contributed equally to this article.

**Keywords:** Influenza, oseltamivir, nursing homes, outbreak control, resistance, prophylaxis, prevention, simulation model, research, expedited

## Abstract

Prophylaxis with neuraminidase inhibitors is important for controlling seasonal influenza outbreaks in long-term care settings. We used a stochastic individual-based model that simulates influenza virus transmission in a long-term care nursing home department to study the protection offered to patients by different strategies of prophylaxis with oseltamivir and determined the effect of emerging resistance. Without resistance, postexposure and continuous prophylaxis reduced the patient infection attack rate from 0.19 to 0.13 (relative risk [RR] 0.67) and 0.05 (RR 0.23), respectively. Postexposure prophylaxis prevented more infections per dose (118 and 323 daily doses needed to prevent 1 infection, respectively) and required fewer doses per season than continuous prophylaxis. If resistance to oseltamivir was increased, both prophylaxis strategies became less efficacious and efficient, but postexposure prophylaxis posed a lower selection pressure for resistant virus strains. Extension of prophylaxis to healthcare workers offered little additional protection to patients.

## Abstract 2

Postexposure prophylaxis can prevent more influenza virus infections among nursing home patients per dose than continuous prophylaxis.

The prophylactic use of neuraminidase inhibitors is a key component of influenza outbreak control in healthcare institutions (*1*,*2*). Based on its proven efficacy in reducing susceptibility, duration of illness, and infectiousness in household studies (*3*–*6*), oseltamivir is now the antiviral agent recommended for prophylactic use in nursing homes. Although the efficacy of oseltamivir has not been extensively assessed in the elderly, some observational and experimental studies suggest beneficial effects of both continuous and postexposure prophylaxis in containing outbreaks and reducing the number of severe complications among nursing home residents (*2*,*7*–*10*).

During the 2007–08 and 2008–09 influenza seasons, the number of isolated influenza A (H1N1) viruses with resistance to the neuraminidase inhibitor oseltamivir increased considerably (*11*,*12*). Following the emerging resistance against the M2-inhibitors amantadine and rimantadine, the efficacy of this class of neuraminidase inhibitors may also be threatened (*13*). Given the speed at which resistant strains have spread and the large variability of influenza activity, it has been impossible to obtain evidence on how resistance has affected influenza control strategies from randomized controlled trials. This effect can, however, be derived using modeling studies (*14*,*15*). Therefore, we developed a mathematical model of influenza transmission in long-term care facilities to study different scenarios and to perform multiple simulations that minimize the probability of chance outcomes. We primarily determined the effect and efficiency of postexposure and continuous exposure prophylaxis strategies with oseltamivir, as compared with no prophylaxis, on infection attack rates among patients in a long-term care nursing home department. We also determined the influence of increased introduction of resistant virus strains on both strategies and assessed the potential benefits of extending prophylaxis to healthcare workers (HCWs).

## Methods

### Population and Model

We simulated the occurrence of influenza virus outbreaks during an 80-day period in a typical long-term care nursing home department (30-bed unit with 15 two-bed rooms and a team of 30 HCWs) in the Netherlands. HCWs worked 8-hour shifts; according to a weekly schedule 5, 3, and 1 HCW(s) worked during the day, evening, and night shifts, respectively, which has been observed in some nursing homes in the Netherlands. The average length of stay for a patient was 14 months (*16*,*17*). Because we simulated a small population where chance events can have major effects, we used a stochastic transmission model. The model is described in the online supporting information (Technical Appendix) and has been described in detail in a previous study (*18*). Here, we describe the essential elements of the model’s structure for the baseline scenario (parameters for the baseline scenario are shown in Table 1).

**Table 1:** Parameter values baseline scenario*

Parameter	Value	Reference
No. beds	30	
No. HCWs	30	
Time step (= shift), h	8	(*18*)
Minimum duration of simulation, d	80	
Discharge/mortality rate, per d	1/425	(*16**,**17*)
Rate of becoming infectious after infection, per d	1/1/4	(*20**,**21*)
Infection recovery rate, per d	1/1/4	(*20**,**21*)
Prior immunity HCWs	0.3	(*22*)
Prior immunity patients	0	
Vaccine uptake patients	75%	(*25*)
Vaccine uptake HCWs	40%	(*2*)
Vaccine efficacy (against infection)		
Patients	25%	(*28*)
HCWs	73%	(*27*)
Transmission probability per casual contact	0.13	(*18*)
Close/casual transmission probability ratio	2	
Mean visitor frequency/patient/d	0.7	(*31*)
Minimum duration of postexposure prophylaxis, d	14	(*2*)
Minimum duration of postexposure prophylaxis after last detected case, d	8	(*2*)
Parameters in uncertainty analyses		
Probability of disease developing after infection (range)	0.5 (0.30–0.7)	(*4*)
Probability of disease developing after infection, during prophylaxis (range)	0.2 (0.05–0.4)	(*4*)
Oseltamivir efficacy against infection (range)	0.53 (0.2–0.8)	(*4*)
Oseltamivir reduction in infectiousness (range)	0.2 (0–0.5)	(*4*)

*HCW, healthcare worker.

### Infection Cycle

**Figure 1:**
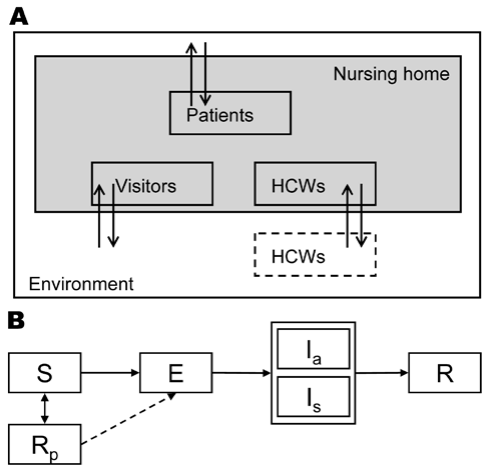
Schematic diagram of our stochastic individual-based model. A) The different types of persons in the nursing home: patients, healthcare workers (HCWs), and visitors. B) The time course of infection: S, susceptible; E, exposed; I_a_, infectious and asymptomatic; I_s_, infectious and symptomatic; R, recovered/immune; R_p_, immune while using prophylaxis. For all patients and HCWs in the model, we kept track of their stage in this infection cycle in time. If the influenza strain that is transmitted is resistant to oseltamivir, persons in the R_p_ department can still become infected (dashed arrow).

According to a standard model for infectious disease transmission, persons could be in 1 of several stages of influenza virus infection: susceptible, infected but not yet infectious (exposed), infectious, or recovered/immune (Figure 1) (*19*). The durations of the exposed and infectious periods were exponentially distributed with means of 1.4 days; the resulting generation time equaled 2.8 days, which agrees with observations of generation times during influenza epidemics (*20*,*21*). At the start of the influenza season, 30% of the adult nursing home population was assumed to be immune to infection because of cross protection from earlier infections (*22*). Since the elderly have weakened immune systems (*23*,*24*), but exact estimates are absent, we made the most conservative assumption that their immune systems had no memory of previous infections.

### Influenza Vaccination

According to our model, both patients and HCWs could receive influenza vaccine before the influenza season. The average vaccination rate was 75% for nursing home patients (*25*) and 40% for HCWs (*2*). We assumed that for each person vaccination either led to perfect immunity against infection or had no effect (*18*). In a previous study, we showed that this all-or-nothing assumption for vaccine-induced immunity yielded similar results to those of an alternative assumption of incomplete immunity in which vaccinated persons had a lower probability of acquiring infection upon contact with an infectious person (*18*). The assumption of all-or-nothing immunity due to prophylaxis has also been made in other modeling studies (*26*). We assumed the vaccine efficacy against influenza virus infection in healthy adults, and thus HCWs, was 73% (*27*). For elderly nursing home patients, no statistically significant vaccine efficacy against infection has been observed (*28*). However, because other evidence showed that the vaccine protected against influenza disease and complications, we assumed patient efficacy to be 25% (*28*,*29*).

### Prophylaxis with Oseltamivir

We compared 2 strategies of prophylaxis with oseltamivir to a control situation in which no neuraminidase inhibitors were used: continuous (seasonal) prophylaxis was given to all patients during 8 weeks (the longest period of prophylaxis described in effectiveness studies) (*30*) around the peak of the influenza season; or postexposure prophylaxis was started for all patients as soon as 1 patient had a laboratory-confirmed influenza virus infection. Because recognition of a possible influenza infection is required before doing a laboratory test, we assumed that only the fraction of infected patients in whom influenza disease developed (the symptomatic patients) could trigger the start of postexposure prophylaxis. We assumed that, for every first symptomatically infected person, the delay between the start of infectiousness and the start of prophylaxis followed a distribution with a mean of 3.5 days. This interval was determined by the time to onset of symptoms, the time to recognition of symptoms, the time to a positive laboratory test, and the delay to start of prophylaxis (Technical Appendix). Postexposure prophylaxis was given to all patients in the department for at least 2 weeks and was continued until no new cases occurred during a period of 8 days (*2*). Because we did not have data on the efficacy of oseltamivir in elderly persons, we used estimates from household studies (*4*) as the best available evidence. We assumed oseltamivir induced immunity to infection by wild-type strains in 55% of the susceptible patients as soon as it was administered and for the duration of prophylaxis. Immunity did not develop in the other patients, but when they were infected they were considered to become less infectious than persons who did not take oseltamivir (*26*). Based on estimates of the total reduction in infectiousness in persons treated with oseltamivir (*4*), we assumed the probability that the virus was transmitted during contact with a susceptible person was reduced by 20%. In the Technical Appendix, we describe some uncertainty analyses that we performed for the parameters describing oseltamivir efficacy.

### Influenza Disease

On the basis of household studies, we assumed that influenza disease would develop in 50% of patients infected with influenza virus (*4*). For those receiving oseltamivir prophylaxis, this probability was only 20% (*4*).

### Contacts

A person’s risk of being infected depended on the number and type of contacts with infectious persons. We distinguished between casual and close contacts; casual contact was considered as conversation and close contact occurred with physical contact. We parameterized the contact model; the expected numbers of contacts, specified by type of persons and kind of contact, matched the number of contacts that we observed in 2 nursing home departments in the Netherlands (*18*). The probability of contact between 2 persons, given their type (HCW or patient), as well as the probability that this contact was close (physical contact), is given in Table 2. During the night shift, patients did not have contact with other patients, except for their roommates, who were assumed to be casual contacts. During the day and evening shifts, patients could also have contact with visitors. All contacts with visitors were considered close. The expected number of visitors was based on a study in the Netherlands on nursing home patients and visitors and was estimated to be 0.7 visitors per patient per day (*31*).

**Table 2:** Contact probabilities between persons in a nursing home department*

Person	Contacted person	Probability of contact	Probability of close contact given casual contact
Patient	Patient	0.07	0.06
Patient	HCW	0.52	0.69
HCW	HCW	0.91	0.31

*HCW, healthcare worker.

### Transmission

For every pair of persons with a casual or close contact, a probability existed that the virus was transmitted if the persons involved in the contact were infectious and susceptible. This probability was determined by sampling from a Bernoulli distribution with mean set equal to the transmission probability. For a casual contact, the transmission probability was 0.13; we chose this probability because the expected infection attack rate among patients in the absence of HCW vaccination was similar to observed attack rates for influenza-like-illness (*18*,*25*,*32*). For close contacts, the probability of transmission was assumed to be 2× as high as that of casual contacts.

### Influenza in the Community

The rate at which influenza virus was introduced into the nursing home by HCWs, visitors, and patients depended on the prevalence of the virus in the community; we used a simulation of an influenza epidemic in a large population (Technical Appendix). In each simulation, a constant proportion of infections in the community was assumed to be caused by resistant strains.

### Oseltamivir Resistance

Resistant viruses were assumed to be completely insensitive to oseltamivir, and therefore prophylaxis had no effect on the susceptibility of a person who was exposed to a resistant strain. We also assumed that use of oseltamivir neither affected the infectiousness nor the development of symptoms in a person infected with a resistant strain. Apart from oseltamivir sensitivity, resistant and nonresistant strains were assumed to be similar. Infection with 1 of the strains conferred cross-protection against infection with other strains during the season.

### Outcomes

We defined the infection attack rate and the disease attack rate as the total number of infections or influenza diseases among patients, respectively, divided by the total number of patients in the nursing home department during the study period. We distinguished between infections caused by oseltamivir-sensitive and -resistant strains and compared scenarios with increasing prevalence of oseltamivir resistance. Based on the distribution of infection attack rates in a nursing home in the absence of preventive measures (*18*), we used the proportion of infection attack rates of >0.3 as a proxy for the probability of a large outbreak. We calculated the absolute and relative risk reductions for both strategies of prophylaxis (efficacy) and determined the fraction of infections caused by resistant strains. We also computed the number of daily doses of prophylaxis needed to prevent 1 infection or disease (DNP) as the total number of doses administered divided by the number of influenza infections or diseases prevented (the absolute risk difference) (efficiency). Information on the statistical precision of the effect estimates can be found in the Technical Appendix.

### Alternative Scenarios

In addition to the baseline scenario previously described, we considered an alternative scenario in which both patients and HCWs received continuous or postexposure prophylaxis according to the same rules. Postexposure prophylaxis was started after detection of infection in a patient and was given to all patients and all HCWs. We also studied a scenario in which the HCW vaccination rate was only 10%, as was observed in the Netherlands (*33*). Here we considered prophylaxis to patients only and to patients and HCWs.

In the Technical Appendix, additional scenarios are described for the following circumstances: 1) different delays between the start of infectiousness of the first symptomatic patient and the start of postexposure prophylaxis, 2) different levels of influenza virus activity in the community, 3) higher percentage of HCWs vaccinated, 4) lower patient vaccine uptake, 5) greater percentage of patients with prior immunity, and 6) a 60-bed nursing home department.

## Results

### Baseline Scenario

**Figure 2:**
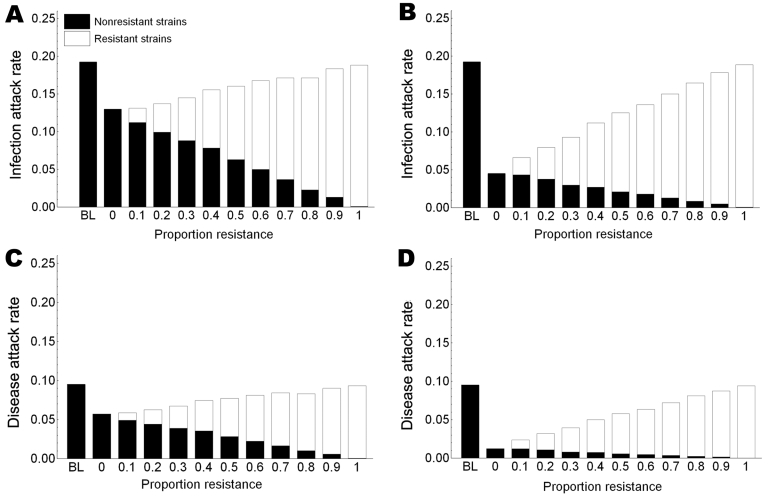
Effects of prophylaxis with oseltamivir on influenza virus infection and disease rates among nursing home patients. The effects of both postexposure and continuous prophylaxis strategies are shown for different proportions of resistant virus strains in the community and compared with a control setting without prophylaxis and resistance. Panels A and C, postexposure prophylaxis given to all patients; panel B and D, continuous prophylaxis for 8 weeks. BL, baseline.

**Figure 3:**
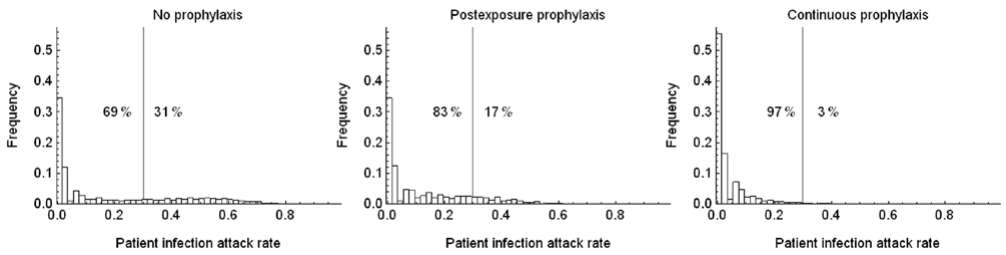
Distribution of influenza virus infection attack rates among patients who received no prophylaxis, postexposure prophylaxis, and continuous prophylaxis in the absence of resistance.

**Figure 4:**
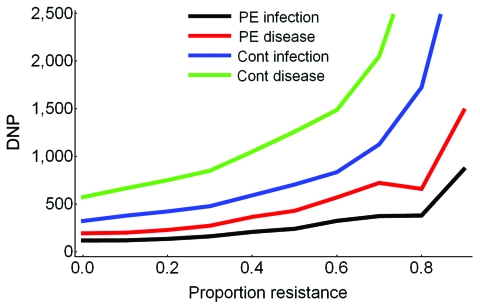
The number of daily doses of oseltamivir needed to prevent 1 influenza virus infection or disease (DNP). Results are shown for both postexposure (PE) prophylaxis and continuous (cont) prophylaxis for increasing proportions of oseltamivir-resistant virus strains in the community.

In the absence of resistance, the prophylactic use of oseltamivir reduced the number of influenza virus infections among patients during the influenza season. The infection attack rate among patients decreased from 0.19 in the control setting without prophylaxis to 0.13 (relative risk [RR] 0.67) when postexposure prophylaxis was given to all patients (first 2 bars, Figure 2, panel A). The fraction of large outbreaks with an infection attack rate of >0.3 decreased from 0.31 to 0.17 (RR 0.55), and outbreaks with attack rates >0.4 rarely occurred (Figure 3). If continuous prophylaxis was given for 8 weeks, the infection attack rate decreased to 0.05 (RR 0.23) (Figure 2, panel B), and the percentage of large outbreaks decreased to 0.03 (RR 0.09). Because of continuous prophylaxis, not only did large outbreaks disappear, but also the percentage of departments without any patient infection increased (Figure 3). Rates of influenza disease decreased from 0.10 to 0.06 (RR 0.60) and 0.01 (RR 0.13), respectively, for the 2 different strategies of prophylaxis (Figure 1, panels C, D). Although the number of infections that could be prevented was higher for continuous prophylaxis, the DNP was ≈3× higher with this strategy than with postexposure strategy (Figure 4). Without resistance, the DNP was 118 for postexposure prophylaxis and 323 for continuous prophylaxis.

### Resistance

**Figure 5:**
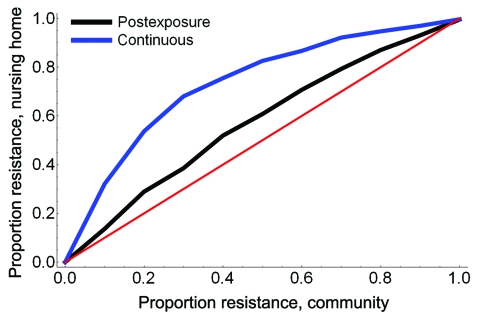
The proportion of infections with oseltamivir-resistant influenza virus strains among nursing home patients for increasing proportions of resistance in the community.

An increase in the proportion of oseltamivir-resistant influenza virus strains in the community reduced the efficacy of prophylaxis with oseltamivir against infection and disease (Figure 2). In addition, both prophylaxis strategies became less efficient and the DNP increased rapidly, in particular for the continuous prophylaxis strategy (Figure 4). Prophylaxis caused a selection pressure for resistant strains; the percentage of infections caused by resistant strains in the nursing home was higher than in the community (Figure 5). The selection of resistant strains was most pronounced for continuous prophylaxis strategy.

### Alternative Scenario: Prophylaxis Extended to HCWs

**Figure 6:**
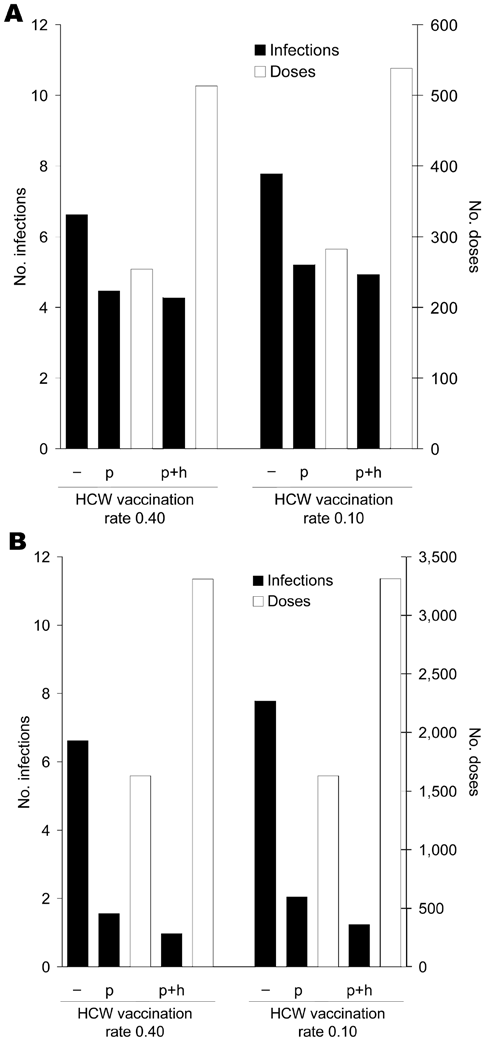
A) Average number of influenza virus infections among patients and B) average number of administered doses of oseltamivir in a 30-bed nursing home department during 1 influenza season. For the postexposure and continuous prophylaxis strategies, results are shown for prophylaxis of patients only (p) and of both patients and healthcare workers (HCWs) (p+h) and compared with a control setting without prophylaxis (–). HCW vaccination rates of 0.4 and 0.1 are considered.

Extension of prophylaxis strategies to include both HCWs and patients offered little additional protection to patients (Figure 6). In the absence of resistance, postexposure and continuous prophylaxis reduced the infection attack rate in HCWs from 0.14 to 0.10 and 0.05, respectively. The attack rate among patients decreased from 0.19 to 0.12 (RR 0.65) and 0.03 (RR 0.15), respectively. Taken together, the DNP for infection (of either patient or HCW) was 140 for postexposure prophylaxis and 366 for continuous prophylaxis; the total number of doses administered was 2 × as high as in the scenario in which only patients received prophylaxis.

When the HCW vaccination rate was 0.1, the infection attack rate among patients without prophylaxis was 0.23. This could be reduced to 0.15 (RR 0.67) when postexposure prophylaxis was given to patients alone and to 0.14 (RR 0.63) when it was given to HCWs as well (Figure 6). Continuous prophylaxis given to patients only or to both patients and HCWs could reduce the infection attack rate to 0.06 (RR 0.26) and 0.04 (RR 0.16), respectively. However, the number of doses required per department was approximately 6 × higher for continuous prophylaxis than for postexposure prophylaxis. Results of other alternative scenarios and the uncertainty analyses are described in the supporting information (Technical Appendix).

## Discussion

Our model predicts that in the absence of resistance, both postexposure prophylaxis and continuous prophylaxis can reduce the number of influenza virus infections in nursing home patients during annual influenza epidemics. Although continuous prophylaxis will prevent more cases, postexposure prophylaxis prevents more cases per dose. If resistance to oseltamivir increases, both prophylaxis strategies become less efficacious and less efficient, with more selection for resistance during continuous prophylaxis. Extension of prophylaxis to HCWs is not expected to have a large effect on the attack rates among patients.

For the results of our modeling study to be correctly interpreted, we must discuss some possible limitations. First, we did not distinguish between different subtypes of influenza circulating in the community. The oseltamivir-resistant strains that dramatically increased in number globally during the last 2 influenza seasons were all influenza A (H1N1) strains and resistance against oseltamivir seemed to be limited to the N1 serotype only. During the 2007–08 season, H1N1 strains were responsible for approximately 60% of influenza virus infections in Europe, which is uncommon when data for the last decade are examined (*34*). The remaining influenza virus infections were caused by A/H3N2 subtype and B type viruses. Thus, even if all influenza A (H1N1) strains acquired resistance against oseltamivir, levels of resistance of >60% are not very probable unless resistance develops as well in the other influenza A subtypes and in influenza B. Second, we did not take into account de novo resistance in persons on prophylaxis. We assumed the probability of emergence of resistance was very low (*26*) and, as we studied a small population, the effect on the outcome was assumed to be negligible. Third, we used estimates on the efficacy of oseltamivir prophylaxis from household studies because we did not have data specific for elderly people. More accurate assessment of efficacy and comparison of preventive measures in nursing homes will require new estimates from studies in senior populations. Finally, we studied a 30-bed department instead of an entire nursing home. If an outbreak occurs in 1 department, it might be necessary to start prophylaxis in other nearby departments as well. However, the effects of prophylaxis for individual departments will not be different.

Our model confirmed the beneficial effects of prophylaxis with oseltamivir in reducing the number of infections and preventing large outbreaks as has been suggested by some observational and experimental studies (*7*–*9*). We have not considered the effects of prophylaxis on the number of complications or deaths, but these can be assumed to be somewhat higher than for infection because oseltamivir also prevents complications when taken after infection (*9**,**35*). Our results suggest a large difference in both efficacy and efficiency between the postexposure and continuous prophylaxis strategies. Although continuous prophylaxis can protect more patients, it also requires large stocks of antiviral drugs and is therefore costly; postexposure prophylaxis might be the preferred strategy. Furthermore, our model suggests that extending prophylaxis to HCWs does not prevent many additional infections among patients when compared with prophylaxis of patients only. Even when the number of infections prevented in HCWs was included, the number of daily doses needed to prevent 1 infection was higher than the number of daily doses needed when prophylaxis was given to patients only. This prediction might be of use for the evaluation of influenza prevention guidelines for nursing homes. Currently, the Dutch guideline for prevention of influenza in nursing homes recommends postexposure prophylaxis for both patients and HCWs (*1*). CDC recommends prophylaxis to nonvaccinated HCWs only, or in case of a mismatch between the vaccine strains and the circulating virus strains, to all HCWs (*2*). Although the latter strategy is expected to be more efficient, the effect on infection attack rates among patients will be less extensive than with prophylaxis of all HCWs. In the postexposure strategy, 1,388 doses of oseltamivir were given to HCWs for every additional prevented infection in a patient. This number was very high compared with the 7 HCW vaccinations needed to prevent 1 infection in patients observed in our previous study (*18*). Therefore, protection of patients by reducing the number of infections in HCWs seems to be more efficiently obtained by increasing vaccine administration among HCWs than by including them in prophylaxis strategies.

Our study suggests that the selection pressure for resistance is lower for postexposure than for continuous prophylaxis. Moreover, the efficiency of postexposure prophylaxis appears to be less sensitive to the level of resistance than that of continuous prophylaxis. During the 2007–08 influenza season, the prevalence of oseltamivir-resistant influenza A (H1N1) strains in Europe increased from <1% in previous years (*11*) to 25% on average, with a national prevalence ranging from 2.5% in Spain up to 66% in Norway (*36*). During the 2008–09 influenza season almost all influenza A (H1N1) strains were oseltamivir resistant (*12*). Oseltamivir use in Europe was low in both years and, in the absence of an apparent selection pressure for resistance, predicting whether resistance will disappear, persist, or increase next season is difficult. Our findings indicate that increasing resistance should be included in the decision-making process for prevention of influenza in healthcare settings. Use of other antiviral agents that are not as associated with resistance should be considered as an alternative prevention strategy (*37*). Household studies suggest that prophylaxis with zanamivir, for example, can give similar results as prophylaxis with oseltamivir (*4*). However, zanamir prophylaxis should be studied in more detail in the nursing home population. Future modeling studies should also address other relevant issues such as the use of combination or cycling therapy approaches (*38*) to retain the protection offered by current antiviral drugs.

## Supplementary Material

Technical Appendix Model and Simulation Algorithms
