# Supplementary material for: A Model-based Assessment of Oseltamivir Prophylaxis Strategies to Prevent Influenza in Nursing Homes
Source: Emerg Infect Dis. 2009 Oct;15(10):1547–55. doi: 10.3201/eid1510.081129 (PMC2866379; doi:10.3201/eid1510.081129)
Supplement: Technical Appendix — Model and Simulation Algorithms [file 08-1129_Techapp-s1.pdf]

A Model-based Assessment of Oseltamivir Prophylaxis Strategies to Prevent Influenza in Nursing Homes

Technical Appendix

Model and Simulation Algorithms

For a detailed description of the model and simulation algorithms we refer to the supporting information in (*1*). We here give the figures referred to in the main text as well as additions and changes to the model structure as described in (*1*).

Influenza in the Community

The rate at which influenza virus was introduced into the nursing home by HCWs, visitors and patients depended on the prevalence of the virus in the community. The spread of influenza in the community of 100,000 individuals outside the nursing home was described by four variables: s, the proportion of susceptible individuals in the community, e, the proportion of exposed individuals in the community, i, the proportion of infectious individuals in the community, r, the proportion of recovered and immune individuals in the community. The daily influenza infection incidence, λ s, and the prevalence, i, in the community (Figure S1) were used in the nursing home model as the hazard rate for HCWs of becoming infected outside the nursing home and the probability that visitors and new patients who entered the nursing home were infectious, respectively. We assumed that during the season a constant fraction φ of all infections was caused by resistant strains (Table S1).

A Stochastic Simulation Model of Influenza Epidemics in a Nursing Home: State Variables

The state of each bed (j=1,2…n) was indicated as x(t,j) and consisted of one variable that took one out of eight possible values: {vacant, susceptible (S), exposed (E), infectious (I), recovered (R), exposed to an oseltamivir resistant virus strain (E_R), infectious with an oseltamivir resistant virus strain (I_R), immune by prophylaxis (R_p)}. The state of each health care worker (HCW) (j=1,2,…m) was indicated as y(t,j) and consisted of two variables. The first variable took one out of two possible values: {at work, not at work}. The second variable took one out of seven possible values: {susceptible (S), exposed (E), infectious (I), recovered (R), exposed to an

oseltamivir resistant virus strain (E_R), infectious with an oseltamivir resistant virus strain (I_R), immune by prophylaxis (R_p)}. At each moment t, the state of the system was completely characterized by the state vectors x and y. For convenience, we also used aggregate variables whose values were completely determined by the state variables:

- the number of patients that were infectious with a non-resistant virus strain at time t, $I_P(t)$;
- the number of HCWs at work that were infectious with a non-resistant virus strain at time t, $I_H(t)$;
- the number of patients that were infectious with an oseltamivir resistant virus strain at time t, I_{RP} (t); the number of HCWs that were infectious with an oseltamivir resistant virus strain at time t, I_{RH} (t).

Update Rules

At each time step Δt the values of the state variables were updated to account for transitions. The probability of each of these transitions to occur was specified according to the rules in Table S2.

Table S1. Parameters in the model

Symbol	Parameter	Default	Units	Ref
n	Number of beds	30		
m	Number of HCWs	30		
Ät	Time step (shift)	8	hours	
T	Minimum duration of simulation	80	days	
ì	Discharge/mortality rate	1/425	day^{-1}	(*2*,*3*)
ó	Rate of becoming infectious after infection	1/1.4	day^{-1}	(*4*,*5*)
ã	Infection recovery rate	1/1.4	day^{-1}	(*4*,*5*)
r_c	Prior immunity HCWs	0.3		(*6*,*7*)
r	Prior immunity patients	0		
u_1	Vaccination rate patients	0.75		(*8*)
u_2	Vaccination rate HCWs	0.4		(*9*)
	Probability of contact between			
c_{11}	Patient – patient	0.07	shift^{-1}	
c_{12}	HCW – patient	0.52	shift^{-1}	
c_{22}	HCW – HCW	0.91	shift^{-1}	
	Probability of close contact between			
ð$_{11}$	Patient – patient	0.06	contact^{-1}	
ð$_{12}$	HCW – patient	0.69	contact^{-1}	
ð$_{22}$	HCW – HCW	0.32	contact^{-1}	
ñ	Close/casual transmission probability ratio	2		
	Vaccine efficacy (against infection)			
ve_1	Patients	0.25		(*10*)
ve_2	HCWs	0.73		(*11*)
p_c	Transmission probability casual contact	0.13	contact^{-1}	(*1*)
g	Average number of visitors	0.7	patient^{-1} day^{-1}	(*12*)
	Minimum duration of post-exposure prophylaxis	14	days	(*9*)
	Minimum duration of post-exposure prophylaxis after last detected case	8	days	(*9*)
ä	Probability of developing disease after infection	0.5		(*13*)
ä$_P$	Probability of developing disease after infection during prophylaxis	0.2		(*13*)
pe_s	Oseltamivir efficacy against infection	0.53		(*13*)
pe_i	Oseltamivir reduction in infectiousness	0.2	contact^{-1}	(*13*)
ö	Fraction of resistant virus strains in the community	0 – 1.0		

Table S2. Transitions and probabilities in the model

	Transition	Probability[#]
Patient flow		
discharge or death	P(x(t+Ät,j) = vacant | x(t,j) = ¬vacant)[¶]	ì Ät
admission	P(x(t+Ät,j) = S | x(t,j) = vacant)	(1 - u_1 ve_1) n ì Ind(P_o (t)=0) Ät + (1 - u_1 ve_1) n ì Ind(P_o(t)=1) (1-pe_s) Ät [*$]
admission	P(x(t+Ät,j) = E | x(t,j) = vacant)	e n ì (1-ö) Ät
admission	P(x(t+Ät,j) = I | x(t,j) = vacant)	i n ì (1-ö) Ät
admission	P(x(t+Ät,j) = R | x(t,j) = vacant)	u_1 ve_1 n ì Ät
admission	P(x(t+Ät,j) = E_R | x(t,j) = vacant)	e n ì ö Ät
admission	P(x(t+Ät,j) = I_R | x(t,j) = vacant)	i n ì ö Ät
admission	P(x(t+Ät,j) = R_p | x(t,j) = vacant)	(1 - u_1 ve_1) n ì Ind(P_o(t)=1) pe_s Ät [*$]
HCW flow		
working	P(y(t+Ät,j) = {at work,·})[‡]	w(t+Ät,j)
at home	P(y(t+Ät,j) = {not at work,·})	1-w(t+Ät,j)
Course of infection of patients		
infection by non-resistant strain	P(x(t+Ät,j)= E | x(t,j) = S)	$ë_1$(t) Ät
becoming infectious from non-resistant strain	P(x(t+Ät,j) = I | x(t,j) = E)	ó Ät
recovery from non-resistant strain	P(x(t+Ät,j) = R | x(t,j) = I)	ã Ät
infection by resistant strain	P(x(t+Ät,j)= E_R | x(t,j) = S)	$ç_1$ (t) Ät
becoming infectious from resistant strain	P(x(t+Ät,j) = I_R | x(t,j) = E_R)	ó Ät
recovery from resistant strain	P(x(t+Ät,j) = R | x(t,j) = I_R)	ã Ät
gain immunity by prophylaxis	P(x(t+Ät,j) = R_p | x(t,j) = S and t=P_{start}) [$]	pe_s
loss of immunity by prophylaxis	P(x(t+Ät,j) = S | x(t,j) = R_p and t=P_{stop}) [$]	1
infection during prophylaxis	P(x(t+Ät,j)= E_R | x(t,j) = R_p)	$ç_1$(t) Ät
Course of infection of HCWs		
infection at work by non-resistant strain	P(y(t+Ät,j) = {·,E} | y(t,j) = {at work, S})	$ë_2$(t) Ät
infection at home by non-resistant strain	P(y(t+Ät,j) = {·,E} | y(t,j) = {not at work, S})	ë s (1-ö) Ät
becoming infectious from non-resistant strain	P(y(t+Ät,j) = {·,I} | y(t,j) = {·,E})	ó Ät
recovery from non-resistant strain	P(y(t+Ät,j) = {·,R} | y(t,j) = {·,I})	ã Ät
infection at work by resistant strain	P(y(t+Ät,j) = {·,E_R} | y(t,j) = {at work, S})	$ç_2$(t) Ät
infection at home by resistant strain	P(y(t+Ät,j) = {·,E_R} | y(t,j) = {not at work, S})	ë s ö Ät
becoming infectious from resistant strain	P(y(t+Ät,j) = {·,I_R} | y(t,j) = {·,E_R})	ó Ät
recovery from resistant strain	P(y(t+Ät,j) = {·,R} | y(t,j) = {·,I_R})	ã Ät
gain immunity by prophylaxis	P(y(t+Ät,j) = {·,R_p} | y(t,j) = {·,S} and t=P_{start}) [$]	pe_s
loss of immunity by prophylaxis	P(y(t+Ät,j) = {·,S} | y(t,j) = {·,R_p} and t=P_{stop}) [$]	1
infection at work during prophylaxis	P(y(t+Ät,j) = {·,E_R} | y(t,j) = (14))	$ç_2$(t) Ät
infection at home during prophylaxis	P(y(t+Ät,j) = {·,E_R} | y(t,j) = {not at work, R_p})	ë s ö Ät

Table S2 (continued)

Forces of infection of non-resistant strains		
For patients		
Day	$ë_1(t) = ë_{11}+ë_{12}+ë_{vis}$	$ë_{11}= (ð_{11}\ ñ +(1-ð_{11}))\ p_c\ c_{11}\ I_P(t)$
Evening	$ë_1(t) = ë_{11}+ë_{12}+ë_{vis}$	$ë_{12}= (ð_{12}\ ñ +(1-ð_{12}))\ p_c\ c_{12}\ I_H(t)$
Night	$ë_1(t) =Ind(x(t,j+Neighbor(j))=I)\ p_c+ë_{12}$ *	$ë_{vis}= g\ ñ\ p_c\ i\ (1-ö)$
For HCWs		
At work	$ë_2(t\ )= ë_{21} +ë_{22}$	$ë_{21}= (ð_{21}\ ñ +(1-ð_{21}))\ p_c\ c_{21}\ I_P(t)$
		$ë_{22}= (ð_{22}\ ñ +(1-ð_{22}))\ p_c\ c_{22}\ I_H(t)$
Forces of infection of resistant strains		
For patients		
Day	$ç_1(t) = ç_{11}+ç_{12}+ç_{vis}$	$ç_{11}= (ð_{11}\ ñ +(1-ð_{11}))\ p_c\ c_{11}\ I_{RP}(t)$
Evening	$ç_1(t) = ç_{11}+ç_{12}+ç_{vis}$	$ç_{12}= (ð_{12}\ ñ +(1-ð_{12}))\ p_c\ c_{12}\ I_{RH}(t)$
Night	$ç_1(t) =Ind(x(t,j+Neighbor(j))=I)\ p_c+ç_{12}$ *	$ç_{vis}= g\ ñ\ p_c\ i\ ö$
For HCWs		
At work	$ç_2(t\ )= ç_{21} +ç_{22}$	$ç_{21}= (ð_{21}\ ñ +(1-ð_{21}))\ p_c\ c_{21}\ I_{RP}(t)$
		$ç_{22}= (ð_{22}\ ñ +(1-ð_{22}))\ p_c\ c_{22}\ I_{RH}(t)$

[#] see table S1 for the meaning on the symbols used
[¶] we use ¬vacant to denote any possible state except vacant
[‡] we use {at work, ·} to denote any possible state where the first state variable is equal to the state at work
* we use Ind(x(t,j)=J) to mean an indicator function that returns the value 1 if its argument is a correct expression, and 0 if its argument is false; we use Neighbor(j)=j-1+2 Mod[j,2] as a function that returns the index of the roommate of the patient, such that Neighbor(1)=2, Neigbor(2)=1
[$] P_{start} and P_{stop} determine the moments of start and end of prophylaxis with oseltamivir; $P_o(t)$= 1 if prophylaxis is being administered.

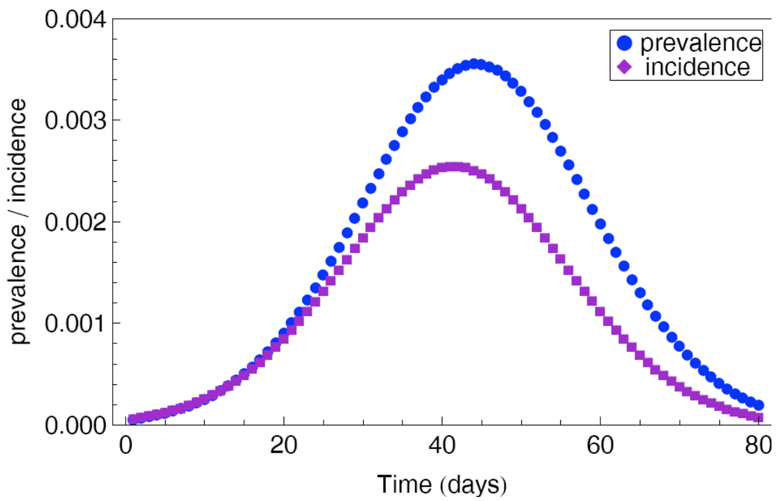

Figure S1. Incidence and prevalence of influenza virus infections in the community.

Prophylaxis with Oseltamivir

Continuous prophylaxis was given during 8 weeks around the peak of the community influenza epidemic, starting from t=15 to t=71. Post–exposure prophylaxis was started for all patients as soon as one patient had a laboratory-confirmed influenza virus infection. Since

recognition of a possible influenza virus infection is required before doing a laboratory test, we assumed only the fraction of infected patients that develop influenza disease (the symptomatic patients) could trigger the start of post-exposure prophylaxis. We assumed that for every first symptomatically infected individual the time between becoming infectious and the start of prophylaxis followed a distribution that was determined by

- the time to onset of symptoms
- the time to recognition of symptoms
- the time to a positive laboratory test
- the time until administration of prophylaxis

For each of these steps the assumed time distributions for the baseline scenario are shown in Figure S2 a to d. The total delay distribution resulting from summation of these steps had a mean delay of 3.5 days (Figure S3a). In Figures S3b and c, distributions of the delay for alternative scenarios are shown, with means of 1.75 and 6 days, respectively. The means and ranges of the duration of the four steps in the different scenarios are shown in Table S3.

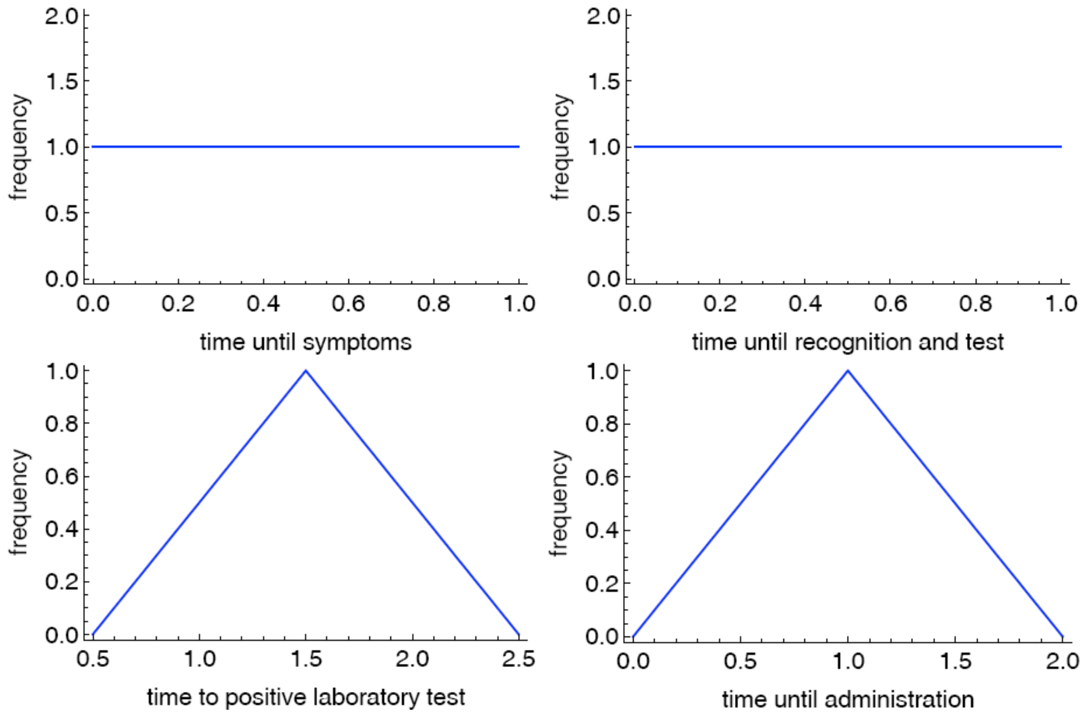

Figure S2. Assumed time distributions for the several steps leading to the delay between the start of infectiousness of the first symptomatically infected individual and the start of post-exposure prophylaxis.

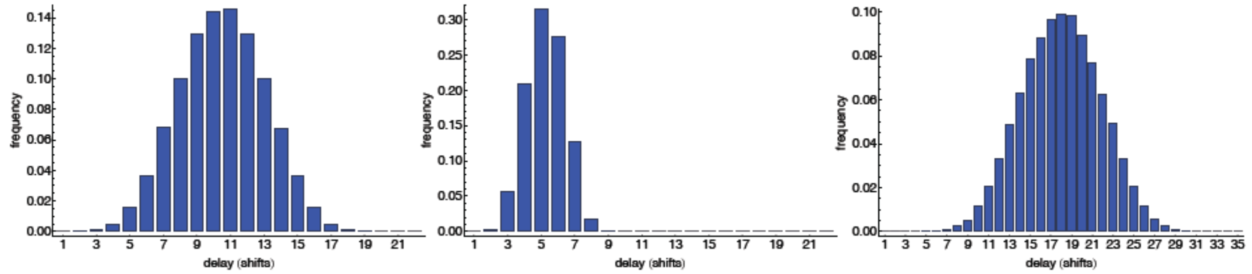

Figure S3. Distributions of the delay between the start of infectiousness of the first symptomatically infected individual and the start of post-exposure prophylaxis with means of 3.5 days, 1.75 days and 6 days, respectively.

Table S3. Mean duration and range of the steps leading to the delay between the start of infectiousness of the first symptomatically infected individual and the start of post-exposure prophylaxis for three scenarios.

Step	Duration (days) Mean (range)	Duration(days) Mean (range)	Duration(days) Mean (range)
Time to onset of symptoms	0.5 (0-1)	0.5 (0-1)	0.5 (0-1)
Time to recognition of symptoms and test	0.5 (0-1)	0.25 (0-0.5)	1 (0-2)
Time to positive laboratory test	1.5 (0.5 -2.5)	0.75 (0.5-1)	2.5 (1.5-3.5)
Time until administration	1.0 (0-2)	0.25 (0-0.5)	2 (0 – 4)
Total	3.5 (0.5-6.5)	1.75 (0-3)	6 (1.5-10.5)

Table S4. Precision of the effect estimates, as mean and 95% bootstrap confidence interval for the baseline scenario based on 4000 simulations, and an alternative scenario (reduced vaccine uptake of patients) based on 2000 simulations.

	RR PE	RR cont	NNT PE	NNT cont
Baseline scenario	0.67 (0.64-0.70)	0.23 (0.22-0.24)	118 (105-135)	323 (309-339)
Patient vaccine uptake 0.4	0.66 (0.62-0.70)	0.24 (0.22-0.26)	97 (82-115)	268 (254-283)

Precision of the Effect Estimates

In the main text we did not give confidence intervals around the effect estimates because these depended on the number of simulations we performed. The 95% bootstrap confidence intervals around the effect estimates (Table S4), show us that the 4000 simulations for the baseline scenario and the 2000 simulations for the alternative scenarios sufficed to obtain reliable effect estimates (Table S4).

Uncertainty analyses

We used Latin hypercube sampling (*15,16*) to do uncertainty analyses for the four parameters describing oseltamivir effectiveness. Therefore we chose a likely range for the parameter values in question and drew actual values from a uniform distribution over this range. For every scenario under study we made 50 different parameter sets such that the whole range of possible values for each of the four parameters was represented equally. We varied the parameter oseltamivir efficacy against infection over a range from 0.2 to 0.8 based upon the reported

confidence intervals (top row), the reduction in infectiousness caused by oseltamivir from 0 to 0.5 (second row), the probability of developing disease (symptom probability) without prophylaxis from 0.3 to 0.7 (third row) and the probability of developing disease during prophylaxis from 0.05 to 0.4 (bottom row), according to the estimated confidence intervals. The results are shown in figure S4 for both post-exposure and continuous prophylaxis. The influenza virus attack rate among patients showed a strong negative correlation with the efficacy of oseltamivir to protect against infection for both strategies of prophylaxis. A less strong correlation was present with the oseltamivir induced reduction in infectiousness. The probability of developing disease was weakly correlated with the patient infection attack rate for the post-exposure, but not for the continuous prophylaxis strategy. The probability of developing disease during prophylaxis did not have a large impact on the attack rate.

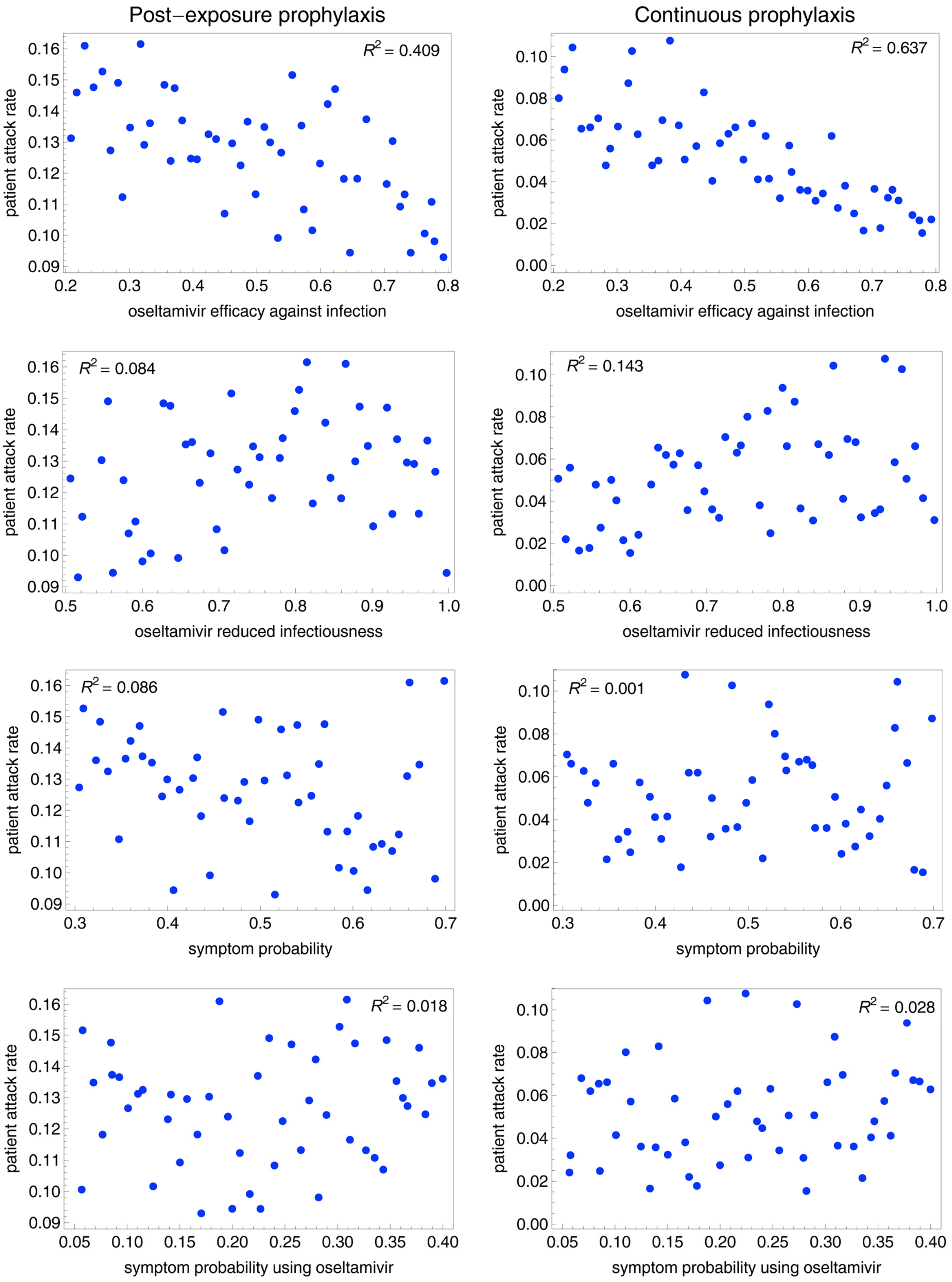

Figure S4. Results of uncertainty analyses. We varied the parameters oseltamivir efficacy against infection, oseltamivir reduction in infectiousness and symptom probability (probability of developing disease) with or without prophylaxis.

Alternative Scenarios

Table S5 shows the results of some additional scenarios:

1) Two scenarios with delays between the start of infectiousness of the first symptomatic patient and the start of post-exposure prophylaxis of 1.75 and 6 days instead of 3.5 days. Changes in the delay did not have a large influence on the results.

2) Two scenarios with higher and lower influenza virus activity in the community (with community attack rates of 15 and 5 percent as compared to 10 percent in the baseline scenario). Apparently the number of doses needed to prevent one infection was not very sensitive to the annual influenza activity when prophylaxis was given post-exposure. With continuous prophylaxis, the number of prevented cases increased with higher influenza prevalence and the strategy became more efficient, although it did not approximate the efficiency of post-exposure prophylaxis.

3) A scenario in which the HCW vaccination rate was 0.75 instead of 0.4. In this scenario the influenza virus attack rate among patients was already decreased in the absence of prophylaxis. Although the relative risk reductions for both strategies of prophylaxis were similar to those in the baseline scenario, the DNP increased due the lower actual number of infections prevented.

4) A scenario in which the patient vaccine uptake was 0.40 instead of 0.75. In this scenario, the infection attack rate was slightly increased in the absence of prophylaxis, which increased the efficiency of prophylaxis.

5) A scenario in which 30% instead of 0% of the patients was immune at the start of the season. In this scenario the effectiveness and efficiency of prophylaxis decreased since more individuals were protected prior to prophylaxis and a lower number of infections was prevented.

6) A scenario for a larger department with 60 beds. In this simulation we assumed the ratio of patients and HCWs and the average number of contacts per person per day to be the same as we had observed in the 30-bed departments. The effect of prophylaxis became slightly higher in this scenario due to the higher attack rate in the absence of prophylaxis.

Table S5. Effects and efficiency of post-exposure prophylaxis (compared with no prophylaxis) in reducing influenza virus infection attack rates among nursing home patients for different scenarios (see text above).

		Mean infection attack rate			*Relative risk*		*Daily doses needed to prevent one infection*	
Scenario		No	Post-exposure	Continuous	Post-exposure	Continuous	Post-exposure	Continuous
0	Baseline	0.19	0.13	0.05	0.67	0.23	118	323
1a	1.75-day delay	0.19	0.11	-	0.59	-	99	-
1b	6-day delay	0.19	0.15	-	0.78	-.	161	-
2a	High virus activity	0.31	0.20	0.08	0.64	0.26	104	206
2b	Low virus activity	0.11	0.08	0.03	0.69	0.26	127	579
3	HCW vaccine uptake 0.75	0.13	0.09	0.03	0.70	0.24	155	471
4	Patient vaccine uptake 0.4	0.23	0.15	0.06	0.66	0.24	97	268
5	Prior immunity patients 0.3	0.10	0.08	0.03	0.74	0.27	218	378
6	60-bed department	0.26	0.13	0.06	0.51	0.21	97	229

References

1. van den Dool C, Bonten MJ, Hak E, Heijne JC, Wallinga J. The Effects of Influenza Vaccination of Health Care Workers in Nursing Homes: Insights from a Mathematical Model. PLoS Med. 2008;5:e200. PubMed DOI: 10.1371/journal.pmed.0050200

2. CTG ZAio. Doelmatigheid verpleeghuizen in relatie tot verantwoorde zorg onderzocht. 21-6-2006. Utrecht, CTG/ZAio.

3. Tekstproducties JCM. Arcares jaarverslag 2005. 2006. Utrecht, Arcares.

4. Hirotsu N, Ikematsu H, Iwaki N, Kawai N, Shigematsu T, Kunishima O, et al. Effects of antiviral drugs on viral detection in influenza patients and on the sequential infection to their family members--serial examination by rapid diagnosis (Capilia) and virus culture. Int Congr Ser. 2004;1263:105–8. DOI: 10.1016/j.ics.2004.02.020

5. Wallinga J, Lipsitch M. How generation intervals shape the relationship between growth rates and reproductive numbers. Proc Biol Sci 2007;274:599–604.

6. Rvachev A, Longini IM. A mathematical model for the global spread of influenza. Math Biosci. 1985;75:3–22. DOI: 10.1016/0025-5564(85)90064-1

7. Cauchemez S, Valleron AJ, Boelle PY, Flahault A, Ferguson NM. Estimating the impact of school closure on influenza transmission from Sentinel data. Nature. 2008;452:750–4. PubMed DOI: 10.1038/nature06732

8. Hayward AC, Harling R, Wetten S, Johnson AM, Munro S, Smedley J, et al. Effectiveness of an influenza vaccine programme for care home staff to prevent death, morbidity, and health service use among residents: cluster randomised controlled trial. BMJ. 2006;333:1241. PubMed DOI: 10.1136/bmj.39010.581354.55

9. Fiore AE, Shay DK, Broder K, Iskander JK, Uyeki TM, Mootrey G, et al. Prevention and control of influenza: recommendations of the Advisory Committee on Immunization Practices (ACIP), 2008. MMWR Recomm Rep. 2008;57:1–60. PubMed

10. Jefferson T, Rivetti D, Rivetti A, Rudin M, Di Pietrantonj C, Demicheli V. Efficacy and effectiveness of influenza vaccines in elderly people: a systematic review. Lancet. 2005;366:1165–74. PubMed DOI: 10.1016/S0140-6736(05)67339-4

11. Jefferson T, Rivetti D, Di Pietrantonj C, Rivetti A, Demicheli V. Vaccines for preventing influenza in healthy adults. Cochrane Database Syst Rev. 2007;CD001269. PubMed

12. de Klerk, M. Ouderen in Instellingen. Landelijk overzicht van de leefsituatie van oudere tehuisbewoners. (7), 26-29. 2005. Den Haag, Sociaal en Cultureel Planbureau.

13. Halloran ME, Hayden FG, Yang Y, Longini IM Jr, Monto AS. Antiviral effects on influenza viral transmission and pathogenicity: observations from household-based trials. Am J Epidemiol. 2007;165:212–21. PubMed DOI: 10.1093/aje/kwj362

14. Backer H. Counterpoint: in favor of mandatory influenza vaccine for all health care workers. Clin Infect Dis. 2006;42:1144–7. PubMed DOI: 10.1086/501463

15. Blower SM, Hartel D, Dowlatabadi H, Anderson RM, May RM. Drugs, sex and HIV: a mathematical model for New York City. Philos Trans R Soc Lond B Biol Sci. 1991;331:171–87. PubMed DOI: 10.1098/rstb.1991.0006

16. Sanchez MA, Blower SM. Uncertainty and sensitivity analysis of the basic reproductive rate - Tuberculosis as an example. Am J Epidemiol. 1997;145:1127–37. PubMed
